# A Self-Healing Polymer with Fast Elastic Recovery upon Stretching

**DOI:** 10.3390/molecules25030597

**Published:** 2020-01-30

**Authors:** Pei-Chen Zhao, Wen Li, Wei Huang, Cheng-Hui Li

**Affiliations:** 1 State Key Laboratory of Coordination Chemistry, School of Chemistry and Chemical Engineering, Nanjing National Laboratory of Microstructures, Collaborative Innovation Center of Advanced Microstructures, Nanjing University, Nanjing 210023, China171870585@smail.nju.edu.cn (W.L.); 2 Shenzhen Research Institute of Nanjing University, Shenzhen 518057, China

**Keywords:** self-healing, PDMS, imine bond, elasticity, polymer

## Abstract

The design of polymers that exhibit both good elasticity and self-healing properties is a highly challenging task. In spite of this, the literature reports highly stretchable self-healing polymers, but most of them exhibit slow elastic recovery behavior, i.e., they can only recover to their original length upon relaxation for a long time after stretching. Herein, a self-healing polymer with a fast elastic recovery property is demonstrated. We used 4-[tris(4-formylphenyl)methyl]benzaldehyde (TFPM) as a tetratopic linker to crosslink a poly(dimethylsiloxane) backbone, and obtained a self-healing polymer with high stretchability and fast elastic recovery upon stretching. The strain at break of the as-prepared polymer is observed at about 1400%. The polymer can immediately recover to its original length after being stretched. The damaged sample can be healed at room temperature with a healing efficiency up to 93% within 1 h. Such a polymer can be used for various applications, such as functioning as substrates or matrixes in soft actuators, electronic skins, biochips, and biosensors with prolonged lifetimes.

## 1. Introduction

Inspired by the self-healing phenomenon in natural biomaterials, many self-healing polymers have been synthesized to repair internal or external damage. These polymers can self-heal damage through a certain mechanism and therefore prolong the service time of materials in various applications [1,2,3,4,5]. The initial strategy for constructing self-healing polymers is based on incorporating healing agents which are blended into the polymer matrix [6,7,8,9]. However, the repairs in such systems can only be repeated for a limited number of times since the encapsulated agents will be depleted after healing. To overcome this limitation, researchers have designed and synthesized new self-healing polymers with reversible chemical bonds (either reversible covalent bonds or dynamic non-covalent interactions). So far, many reversible covalent bonds, such as Diels–Alder (DA) reactions [10,11,12], [2 + 2] cycloaddition [13,14], acylhydrazone bonds [15,16], trithiocarbonate units [17,18], disulfide moieties [19,20,21], diarylbibenzofuranone [22,23], and imine bonds [24,25], or non-covalent interactions, such as hydrogen bonds [26,27,28,29], hydrophobic interactions [30], π…π stacking interactions [31,32,33], ionic interactions [34,35] and metal–ligand interactions [36,37,38,39,40,41,42,43,44], have been incorporated into various kinds of polymers as cross-linkages, leading to repeatable self-healing polymers.

Elastic polymers have been used in a myriad of products, from hoses and cables to adhesives and clothing. Endowing elastic polymers with self-healing features is therefore of great economic importance. However, synthetic polymers often face a trade-off problem between elasticity and self-healing ability. Typically, elastomers are composed of three-dimensional networks of long polymer chains [45]. The elasticity is derived from the ability of the long chains to reconfigure themselves under an applied tension. When the tension is moved, the strong covalent cross-linking sites ensure that the cross-linked networks can recover to their original configuration. Thus, if the cross-linking bonds in polymers are not strong enough, they will be disassociated easily upon stretching, resulting in a permanent deformation. However, weak dynamic bonds are often presented as cross-linking sites for many autonomously self-healing materials. These cross-linking bonds will break upon damage and reform after healing [26,27,28,29,30,31,32,33,34,35,36,37,38,39,40,41,42,43,44]. If the cross-linking bonds are too strong, they generally do not show dynamic exchange behavior, and thus polymers based on these bonds cannot self-heal. Therefore, the design of polymers with both good elasticity and self-healing abilities is highly challenging.

Many strategies have been proposed to resolve this conundrum. Guan and colleagues designed stretchable self-healing materials by using a multiphase strategy, in which the stretchability of the materials is conferred by the hard phase while the autonomous self-healing ability is provided by the soft matrix with the multivalent supramolecular interactions [46,47]. Suo and colleagues designed many double-network gels that exhibited both high stretchability and self-healing properties [48,49,50]. Our group and others also designed a series of highly stretchable polymers by elaborately tuning the thermodynamic and kinetic parameters of coordination complexes [37,38,40,44]. However, most of the reported polymers exhibit slow elastic recovery behavior, i.e., they can only recover to their original length after relaxation for a long time upon stretching.

Recently, we reported a polydimethylsiloxane elastomer (BTA-PDMS-25000) which is crosslinked by tetra-functional biphenyl units via imine bonds [51]. The BTA-PDMS-25000 polymer exhibits an impressive self-healing ability (efficient healing within 60 s at room temperature) and high stretchability (elongation > 8000%). However, this polymer is predominantly viscoelastic with significant energy dissipation characteristics. It is desirable to develop new self-healing polydimethylsiloxane elastomers with faster elastic recovery upon stretching. According to the literature, the geometries of cross-linkers have a pronounced effects on the thermal and mechanical properties of polymers [52,53,54]. We therefore envisage that if the planar cross-linker in BTA-PDMS-25000 was substituted by a non-planar one, PDMS polymers with different mechanical and self-healing properties would be readily obtained. For this consideration, 4-[tris(4-formylphenyl)methyl]benzaldehyde (TFPM), which is a tetrahedral crosslinking unit and has been frequently used in covalent organic frameworks [55,56,57], is a good choice. 

In this work, we used 4-[tris(4-formylphenyl)methyl]benzaldehyde (TFPM) as a tetratopic linker to crosslink a poly(dimethylsiloxane) backbone, and obtained a highly stretchable self-healing polymer with fast elastic recovery upon stretching. The strain at break of the as-prepared polymer is observed at about 1400%. The polymer can immediately recover to its original length after being stretched. At room temperature, the damaged samples can be restored after healing for 1 h, with a healing efficiency up to 93%. Such polymers can be used for various applications, such as functioning as substrates or matrixes in soft actuators, electronic skins, biochips, and biosensors with prolonged lifetimes.

## 2. Results and Discussion

### 2.1. Synthesis and General Characterization 

**Figure 1:**
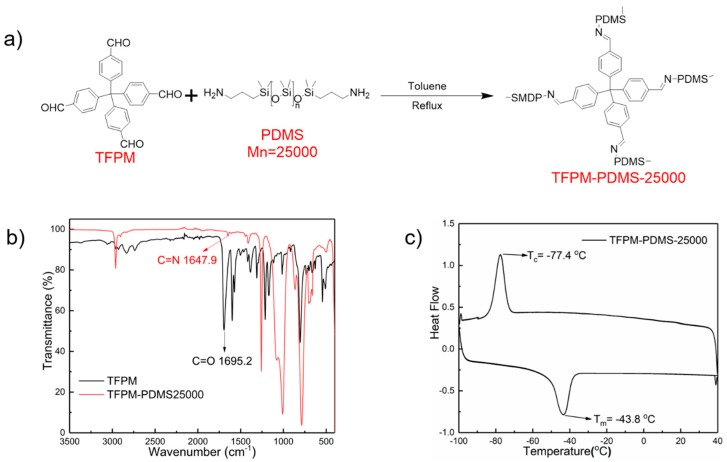
(**a**) Synthesis route of TFPM-PDMS-25000 polymer. (**b**) FTIR spectra of TFPM (4-[tris(4-formylphenyl)methyl]benzaldehyde) and TFPM-PDMS-25000. (**c**) The differential scanning calorimetry (DSC) curve of TFPM-PDMS-25000 polymer.

TFPM-PDMS-25000 was synthesized through a Schiff base condensation reaction between bis(3-aminopropyl)-terminated poly(dimethylsiloxane) (H_2_N-PDMS-NH_2_, M_n_ = 25,000, denoted as PDMS-25000) and TFPM with a molar ratio of 2:1 (Figure 1a). The polymer, which contains polyaldehyde-based molecules as cross-linking points, was synthesized according to the method described in our previous work [51]. The reaction conditions, such as solvent, reaction time, and temperatures can be found in the Experimental section.

The TFPM-PDMS-25000 polymer was characterized by FTIR measurements (Figure 1b). A distinctive C=O group absorption band can be seen in the FTIR spectrum of TFPM at 1695 cm^−1^. After reaction of TFPM and PDMS-25000 at 125 °C for 48 h, the original C=O group absorption band finally disappeared and a new absorption band characteristic for a C=N stretch appeared at 1647.9 cm^−1^. These results indicate that the dynamic polyimine network has been formed. 

We studied the thermal properties of TFPM-PDMS-25000 with differential scanning calorimetry (DSC) and thermal gravimetric analysis (TGA). As shown in Figure 1c, the exothermal peak at −77.4 °C and endothermic peak at −43.8 °C in the DSC curves correspond to crystallization and melting, respectively. The glass transition temperature (*T*_g_) of TFPM-PDMS-25000 polymer must be below −90 °C as there is no other exothermal/endothermal signal between the crystallization point and the lowest measurable temperature of our instrument, indicating that the TFPM-PDMS-25000 polymer is in an elastic state at room temperature (>*T*_g_) and has high chain mobility. The result from the TGA test demonstrates that TFPM-PDMS-25000 has good thermal stability (*T*_d_ = 402 °C, corresponding to 10% weight loss, Figure S1).

### 2.2. Rheological Studies

**Figure 2:**
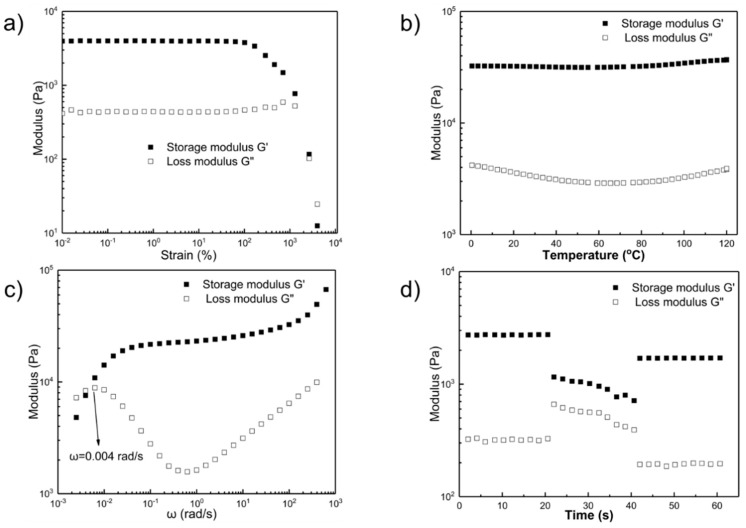
(**a**) Oscillatory strain sweeps of TFPM-PDMS-25000 at different temperatures. (**b**) Dynamic oscillatory temperature sweeps of TFPM-PDMS-25000 ranging from 0 °C to 120 °C at 1 Hz. (**c**) Frequency sweeps of TFPM-PDMS-25000 ranging from 0.001 rad/s to 628 rad/s with 0.1% strain amplitude at room temperature. (**d**) Continuous step strain measurements of TFPM-PDMS-25000 at 25 °C and 1 Hz, under a small strain amplitude 0.1% or a large strain amplitude 1000%.

We used a rotational rheometer to systematically examine the rheological properties of the TFPM-PDMS-25000 polymer. Oscillatory strain sweeps of TFPM-PDMS-25000 showed that the storage modulus G’ (42.5 kPa) was about 10-fold higher than the loss modulus G″ (4.4 kPa) when the strain was less than 1000% at 25 °C (Figure 2a), suggesting that the TFPM-PDMS-25000 polymer is predominantly elastic even at large strains. When the strain was increased (>1000%), the polymer network had been partially broken and led to the sample destruction, as indicated by the sudden decrease of G’ and G″.

To investigate the dynamic temperature-dependent mechanical properties of the TFPM-PDMS-25000 polymer, we performed dynamic oscillatory temperature sweeps from 0 °C to 120 °C at 1 Hz. As presented in Figure 2b, the G’ of TFPM-PDMS-25000 was almost kept constant at this temperature range. No intersection between G’ and G″ was observed in the temperature range from 0 °C to 120 °C, demonstrating the absence of gel-sol transformation. These results suggest that the three-dimensional cross-linking interactions in the TFPM-PDMS-25000 polymer are stable up to 120 °C.

Moreover, we performed frequency sweeps of TFPM-PDMS-25000 polymer films from 0.001 to 628 rad s^−1^ at 25 °C. As shown in Figure 2c, the crossover angular frequency ω_c_ of TFPM-PDMS-25000 is 0.004 rad s^−1^. According to the relationship between characteristic relaxation times τ_c_ and ω_c_ (τ_c_ = 1/ω_c_), the calculated relaxation time τ_c_ is 250 s. The relative short relaxation time is an evidence of quick chain mobility and dynamic exchange of imine bonds in the network of the TFPM-PDMS-25000 polymer.

To investigate the recovery after mechanical breakdown, we conducted continuous oscillation-step strain experiments. As shown in Figure 2d, G’ decreased when the strain increased from 0.1% to 1000%, implying partial breakage of the polymer network. When we set back the strain amplitude to 0.1%, G’ partially recovered but G″ became even smaller, indicating that there are some broken crosslinking bonds in the polymer that remain unrecovered within this time period.

### 2.3. Mechanical Property

**Figure 3:**
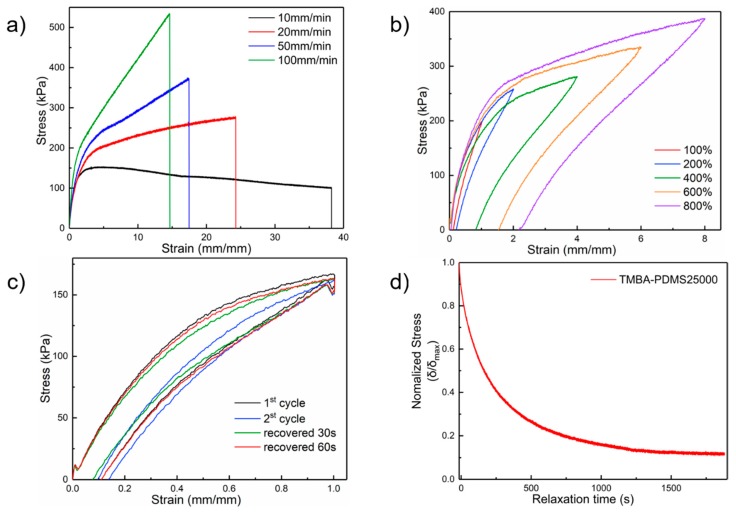
(**a**) Stress–strain curves of the TFPM-PDMS-25000 samples under different stretch speeds ranging from 10 to 100 mm min^−1^ at 25 °C. (**b**) Cyclic stress–strain tests of TFPM-PDMS-25000 with different strains at room temperature. Strain rate = 100 mm min^−1^. (**c**) Cyclic stress–strain tests of TFPM-PDMS-25000 with different relaxation times at room temperature. Strain rate = 100 mm min^−1^. (**d**) Stress relaxation curves of TFPM-PDMS-25000 that was primarily stretched to 100% strain and then allowed to relax for 1800 s at 25 °C.

The mechanical properties of TFPM-PDMS-25000 polymer were investigated by uniaxial tensile tests under different conditions. As illustrated in Figure 3a, the stretchability of this polymer is in correlation with the stretching rate. When we increased the strain speed from 10 to 100 mm min^−1^, the maximum tensile strength of the TFPM-PDMS-25000 polymer increased from 153.7 ± 9.3 kPa to 481.1 ± 8.4 kPa, while the ultimate elongation break decreased from 3800% to 1400% ± 10%. From the low-strain region of the stress–strain curves, the Young’s modulus of TFPM-PDMS-25000 was calculated to be 219.7 ± 8.2 kPa. These data show that TFPM-PDMS-25000 has relatively good mechanical strength and high stretchability.

Next, we performed cyclic stress–strain tests of TFPM-PDMS-25000 under different strains. The loading–unloading curves at 100%, 200%, 400%, 600%, and 800% strain, respectively (Figure 3b), show insignificant hysteresis as compared to other highly stretchable self-healing polymers [37,38,41,42], indicating that TFPM-PDMS-25000 has good elastic recovery. The energy-absorbing efficiencies (ω) are 23.4% to 49.7%, from 100% strain to 800% strain (Table S2), according to the calculation from the integrated area of loading strain–stress curves W1 and unloading strain–stress curves W2. These numbers are quite small among self-healing polymers [37,38,41,42], indicating insufficient energy dissipation during stretching. Compared with the original cycle, a distinct hysteresis loop was observed in the second stretch. However, no significant hysteresis was observed in the subsequent three cycles (Figure S2). Moreover, as shown in Figure 3c, if the same sample was allowed to rest for only 1 min and then tested again, an almost full self-recovery of its original dissipated energy can be observed. When the sample was stretched by hand to about 500% elongation and then released, it recovered as quickly as a rubber (see Video S1 in the Supporting Information). When the sample was compressed, similar fast recovery behavior was observed (Figures S3 and S4, Video S2).

Figure 3d depicts the stress relaxation curves of TFPM-PDMS-25000 film at 25 °C. It can be seen that the normalized stress of TFPM-PDMS-25000 decreased slowly. It takes over 1800 s for the polymer to release 90% of the internal stress (from 1 to 0.1), much longer than the previously reported stretchable self-healing polymers [51]. This observation further demonstrates that the TFPM-PDMS-25000 film has an excellent elastic recovery.

### 2.4. Self-Healing Properties

**Figure 4:**
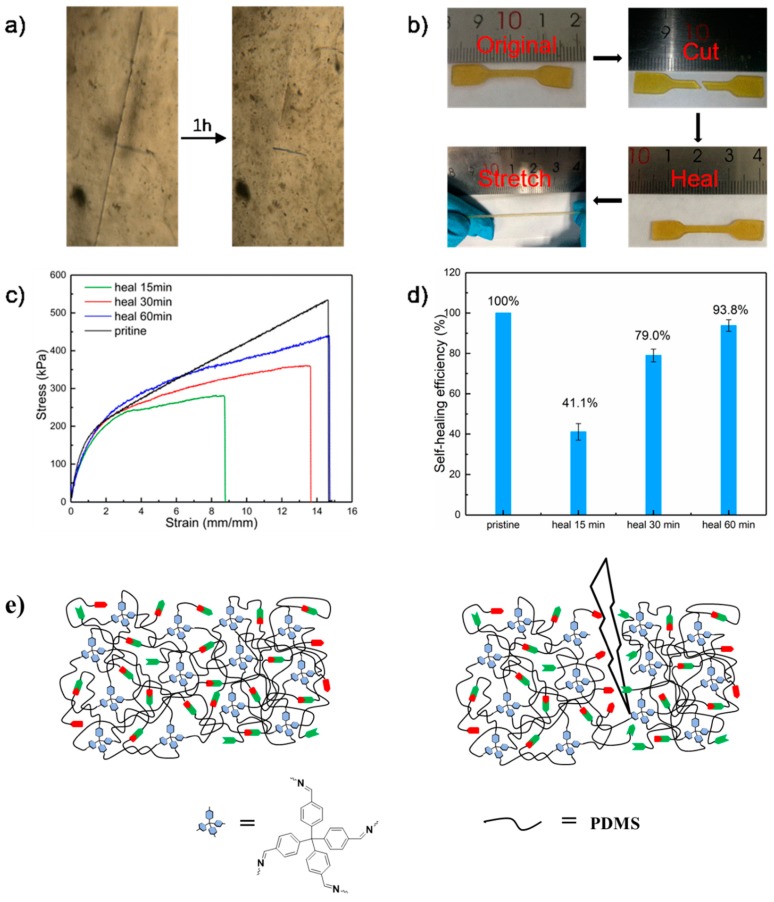
(**a**) Photographs illustrating the macroscopic cutting-healing-stretching procedure of TFPM-PDMS-25000 films at 25 °C. (**b**) Photographs illustrating the macroscopic cutting-healing-stretching procedure of TFPM-PDMS-25000 films at 25 °C. (**c**) Uniaxial tensile tests and (**d**) self-healing efficiencies of TFPM-PDMS-25000 samples for different times at room temperature. (**e**) The possible mechanism for self-healing of the TFPM-PDMS-25000 polymer.

The TFPM-PDMS-25000 elastomer also exhibits impressive self-healing properties under ambient conditions (25 °C, 60 ± 5% Relative Humidity) due to the rupture and reformation of dynamic imine bonds. A TFPM-PDMS-25000 polymer film (thickness ≈ 1.5 mm) was separated into two pieces using a razor blade and then the pieces were put in contact with each other. We used an optical microscope to record the cutting and healing procedures. As shown in Figure 4a, after healing for 1 h at ambient temperature (25 °C), the notch on the TFPM-PDMS-25000 film totally disappeared.

The representative stress–strain curves of the TFPM-PDMS-25000 polymer film healing for different times are shown in Figure 4b. The mechanical self-healing efficiency (η) was calculated from the ratio between the toughness of the healed sample and the toughness of the original sample. In detail, the self-healing efficiency of the TFPM-PDMS-25000 film was 41.1% ± 5.4% after healing for 15 min. It took 1 h to completely heal the cut of the sample with an elongation of 1450% (η = 93.8% ± 3.3%) at room temperature (Figure 4c,d).

The process of self-healing can even happen in 0 °C with a healing efficiency of 79.0% ± 3.9% after 2 h (Figures S5 and S6). Moreover, we can also merge two undamaged polymer films through self-healing ability. As shown in Figure S5, the polymer film was cut into two pieces and then placed in contact with each other via their undamaged surfaces (Figure S7). These results suggest that the imine bonds in the polymer matrix are dynamically exchanging at room temperature (Figure 4e).

### 2.5. Mechanism for the Fast Elastic Recovery

**Figure 5:**
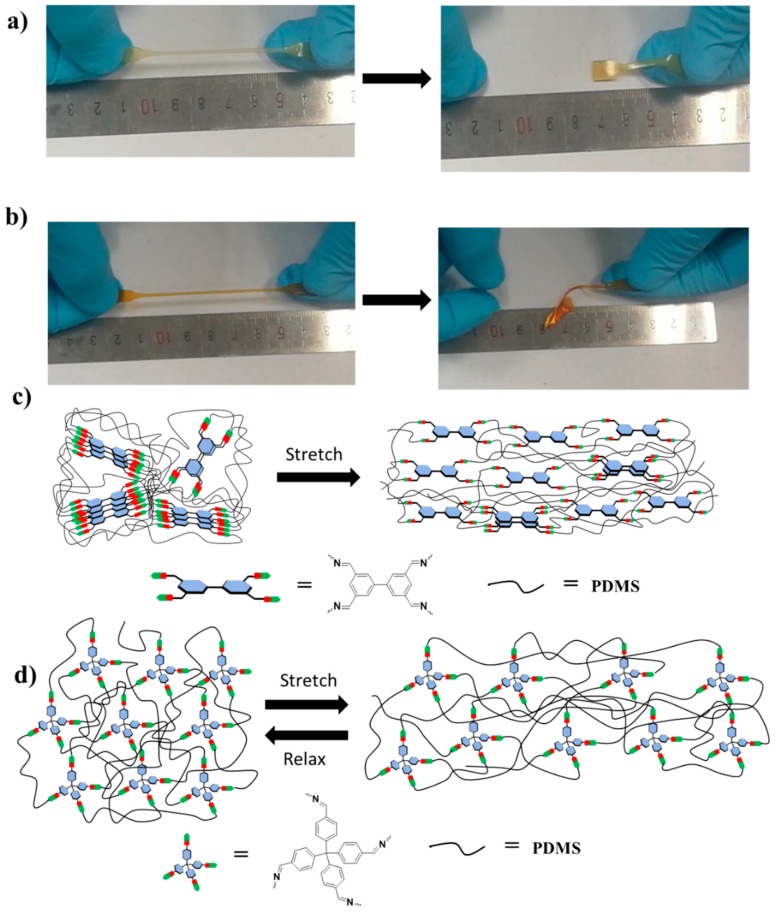
(**a**) Photographs illustrating the stretching and recovery of BTA-PDMS-25000. (**b**) Photographs illustrating the stretching and deformation of TFPM-PDMS-25000. (**c**) The possible mechanism of the stretching and recovery of BTA-PDMS-25000. (**d**) The possible mechanism of the stretching and recovery of TFPM-PDMS-25000.

In our recent work, we reported a polydimethylsiloxane elastomer (BTA-PDMS-25000) which is also crosslinked by tetra-functional biphenyl units via imine bonds [51]. The BTA-PDMS-25000 polymer exhibits an impressive self-healing ability (efficient healing within 60 s at room temperature) and high stretchability (elongation > 8000%). However, this polymer is predominantly viscoelastic with significant energy dissipation characteristics. As shown in Video S1 and Figure 5a,b, in contrast to TFPM-PDMS-25000, the shape of BTA-PDMS-25000 cannot be quickly recovered after stretching. The only difference between these two polymers is that the crosslinking unit is planar in BTA-PDMS-25000 but non-planar in TFPM-PDMS-25000. We therefore speculate that the spatial configuration of the crosslinking unit plays a critical role in the mechanical properties. 

It is known that elasticity in polymers originated from the reconfiguration of the chains under an applied tension. If there is viscous deformation and/or bond breakage presented during stretching, then the network will need a longer time to return to its original configuration or it cannot recover when the tension is removed. In BTA-PDMS-25000, the PDMS chains are cross-linked by planar aromatic tetra-functional biphenyl units. The crosslinking units tend to stack together through π…π interactions. The dynamic π…π interactions will be broken and re-formed during stretching, leading to sliding of the polymer chains which cause permanent deformation (Figure 5c). In contrast, for TFPM-PDMS-25000, the polymer forms a random three-dimensional network of interconnected chains due to the tetrahedral spatial configuration of tetraphenylmethane units, making it more difficult to flow between polymer chains (Figure 5d). The stretching of the random coil polymer chains in TFPM-PDMS-25000 is reversible, leading to the fast elastic recovery. Moreover, the reduced polymer chain mobility retards the self-healing process.

## 3. Experimental Section

### 3.1. Materials

Bis(3-aminopropyl) terminated poly(dimethylsiloxane) (H_2_N-PDMS-NH_2_, M_n_ = 25,000, noted as PDMS-25000) was obtained from Gelest. TriphenylMethyl chloride, aniline, isoamyl nitrite, hypophosphorous acid, *n*-Butyllithium (2.5 mol/L in hexane) concentrated sulfuric acid bromine, and the other reagents and solvents were used without further purification after purchasing from Sigma-Aldrich (St. Louis, MO, USA). TFPM was synthesized using the method presented in the literature [55].

### 3.2. General Measurements

FTIR spectra were tested with a Bruker (Billerica, MA, USA) Tensor 27 Fourier-transform infrared spectrometer. Thermal gravimetric analysis (TGA) data were performed on a PerkinElmer TA 2100-SDT 2960 (Waltham, MA, USA) under a N_2_ atmosphere. The temperature range and heating rate for TGA measurement were 30–1000 °C and 10 °C min^−1^, respectively. Differential scanning calorimetry (DSC) experiments were executed on a DSC apparatus of Mettler-Toledo (Zurich, Switzerland) with temperatures ranging from −100 to 40 °C and heating/cooling speed of 10 °C min^−1^ under a nitrogen atmosphere. Temperature and enthalpy calibrations were performed before the experiments using zinc and indium standards. Furthermore, each sample was tested for three cooling-heating runs and the data were procured from the second cooling-heating curves. Optical microscopy images were recorded by Nikon ECLIPSE E100 optical microscope (Tokyo, Japan).

### 3.3. Preparation of TFPM-PDMS-25000 Polymer Films

Under a N_2_ atmosphere, TPFM (86.5 mg, 0.2 mmol) and H_2_N-PDMS-NH_2_ (10.00 g, M_n_ = 25,000) was added to in redistilled toluene (200 mL) with constant stirring. The reaction mixture was heated to reflux with an oil bath for 24 h. Then the reaction was cooled down to RT and toluene was partially removed by evaporation under reduced pressure. Then, the remaining part was poured into a polytetrafluoroethylene (PTFE) mold by drying in vacuo (at 90 °C, 24 h). The TFPM-PDMS-25000 polymer films were then taken out from the PTFE mold and cut into certain dimensions for further testing.

### 3.4. Rheological Test

The rheological measurements were performed on a TA DHR-2 Rheometer (DHR-2, TA Instruments, New Castle, DE, USA). A 20 mm parallel plate with circular samples of 20 mm diameter was used during the tests. The gap distance was set to 1000 μm. Contact force with the sample was set to 0.20 ± 0.15 N and maintained by the auto-compression feature. Oscillatory strain sweeps were conducted at 25 °C and 1 Hz with stain ranging from 0.01% to 1000%. Temperature sweeps were performed from 0 °C to 100 °C with a sweeping rate of 5 °C min^−1^ and a frequency of 1 Hz. In order to keep the measured torque at a reasonable value when the sample was softened, the strain was automatically modulated at 0.10% ± 0.02% by the instrument. Frequency sweeps were run from 0.001 to 628 rad s^−1^ with a constant 0.1% strain amplitude at 25 °C.

### 3.5. Mechanical and Self-Healing Measurements

Uniaxial tensile experiments were conducted at room temperature and on an Instron 3343 (Boston, MA, USA) instrument equipped with a 500 N load cell. For all the tests, a sample size of 50 mm length × 5 mm width × 1 mm height was adopted, and each experiment was carried out three times. For the self-healing tests, two completely separate pieces were obtained by cutting the film. The two pieces were then put together and healed at room temperature. The typical strain–stress curves were obtained by measuring the healed samples in the same procedure as the original samples. The strain rate of self-healing tests is 100 mm min^−1^. Maximal stress strengths, breaking strains, and healing efficiencies (η) are presented as the means ± standard deviation according to the data from at least four trials.

## 4. Conclusions

In summary, we synthesized a new self-healing polymer through one-pot aldimine polycondensation. The as-prepared TFPM-PDMS-25000 polymer shows high stretchablity (elongation >1400%) and fast elastic recovery behavior. This polymer also exhibits excellent self-healing properties and can self-heal within 1 h at room temperature. Such a polymer can be used to prolong the lifetimes of materials in many fabrications, such as sealants, adhesives, coatings, and substrates or matrixes for flexible electronic devices. In addition, our results suggest that optimizing the configuration of crosslinking units of a polymer can lead to a combination of elasticity and self-healing properties in a single polymer, which is helpful for further design of self-healing elastomers.

## Supplementary Materials

The following are available online: Figure S1: The TGA curve of TFPM-PDMS-25000, Figure S2: Five successive loading-unloading cycles of the film prepared from TFPM-PDMS-25000, Figure S3: The successive loading-unloading cycles of the samples of TFPM-PDMS-25000 and BTA-PDMS-25000 in compression tests, Figure S4. Cyclic stress-strain tests of TFPM-PDMS-25000 in compression tests with different relaxation times, Figure S5: Stress-strain curves of TFPM-PDMS-25000 films healing for different times at 0 °C, Figure S6: Healing efficiencies of TFPM-PEA-25000 with different healing durations at 0 °C, Figure S7: Photographs illustrating the macroscopic cutting-healing-stretching procedure of TFPM-PDMS-25000 films at 25 °C, Figure S8: The energy-absorbing properties of TFPM-PDMS-25000 polymer at 100% strain, Table S1: Key mechanical properties of TFPM-PDMS-25000 Film (100 mm min^−1^), Table S2: The energy-absorbing efficiency ω of TFPM-PDMS-25000 for different strains at room temperature, Video S1: The fast elastic recovery experiment of TFPM-PDMS-25000, Video S2: The plastic deformation of TFPM-PDMS-25000 after 30 min of compression, Video S3: The stretching and deformation of BAT-PDMS-25000.
