# Supplementary material for: A Self-Healing Polymer with Fast Elastic Recovery upon Stretching
Source: Molecules. 2020 Jan 30;25(3):597. doi: 10.3390/molecules25030597 (PMC7037885; doi:10.3390/molecules25030597)
Supplement: Supplementary file 1 [file molecules-25-00597-s001.zip › Supplementary Files/Supporting information-633769-Revised.docx]

Electronic Supplementary Information

**A Self-healing Polymer with Fast Elastic Recovery Upon Stretching**

Pei-Chen Zhao ^1^, Wen Li ^1^, Wei Huang ^1, 2, *^ and Cheng-Hui Li ^1,*^

^1^State Key Laboratory of Coordination Chemistry, School of Chemistry and Chemical Engineering, Nanjing National Laboratory of Microstructures, Collaborative Innovation Center of Advanced Microstructures, Nanjing University, Nanjing 210093, P. R. China. [250676625@qq.com](mailto:250676625@qq.com) (P.-C. Z.); [171870585@smail.nju.edu.cn](mailto:171870585@smail.nju.edu.cn) (W. L.);

^2^Shenzhen Research Institute of Nanjing University, Shenzhen, Guangdong Province, 518057, P. R. China.

*Corresponding author:

E-mail: [whuang@nju.edu.cn](mailto:whuang@nju.edu.cn) (W. H.); [chli@nju.edu.cn](mailto:chli@nju.edu.cn) (C.–H. Li)


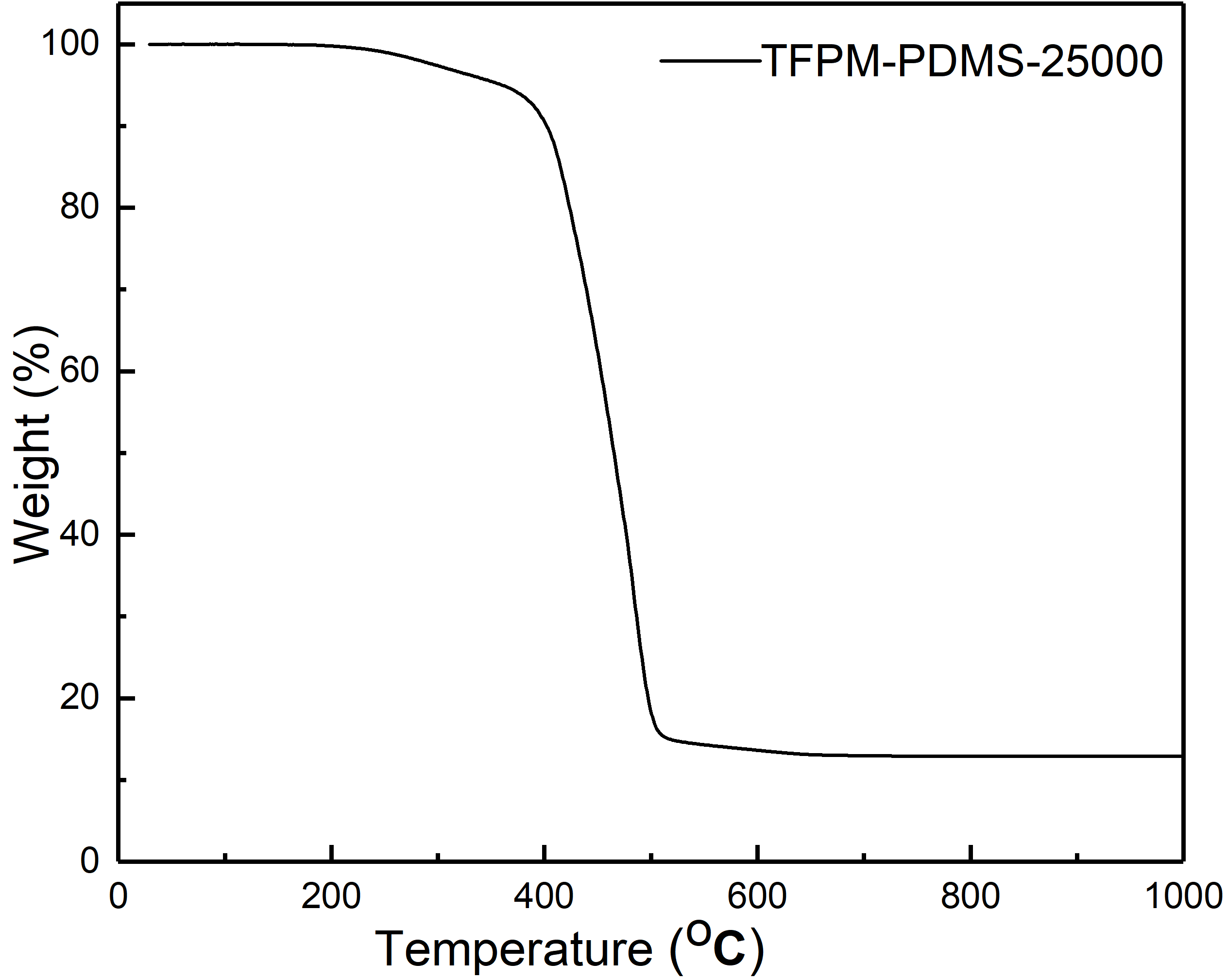


**Figure** **S1**. The TGA curve of TFPM-PDMS-25000.


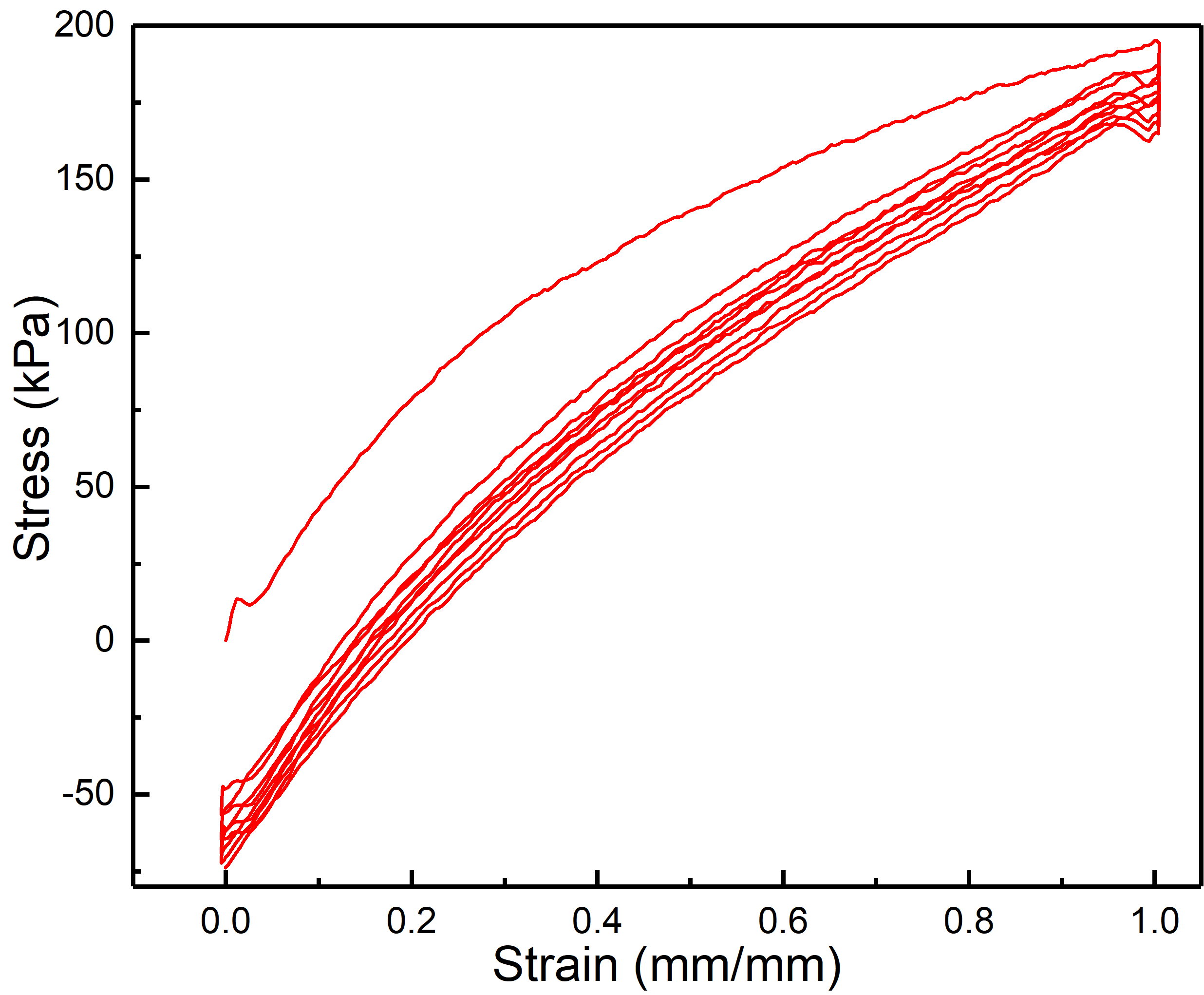


**Figure** **S2**. Five successive loading-unloading cycles of the film prepared from TFPM-PDMS-25000, 100mm min^-1^.





**Figure** **S3.** The successive loading-unloading cycles of the samples of TFPM-PDMS-25000 and BTA-PDMS-25000 in compression test.





**Figure S4.** Cyclic stress-strain tests of TFPM-PDMS-25000 in compression test with different relaxation times.


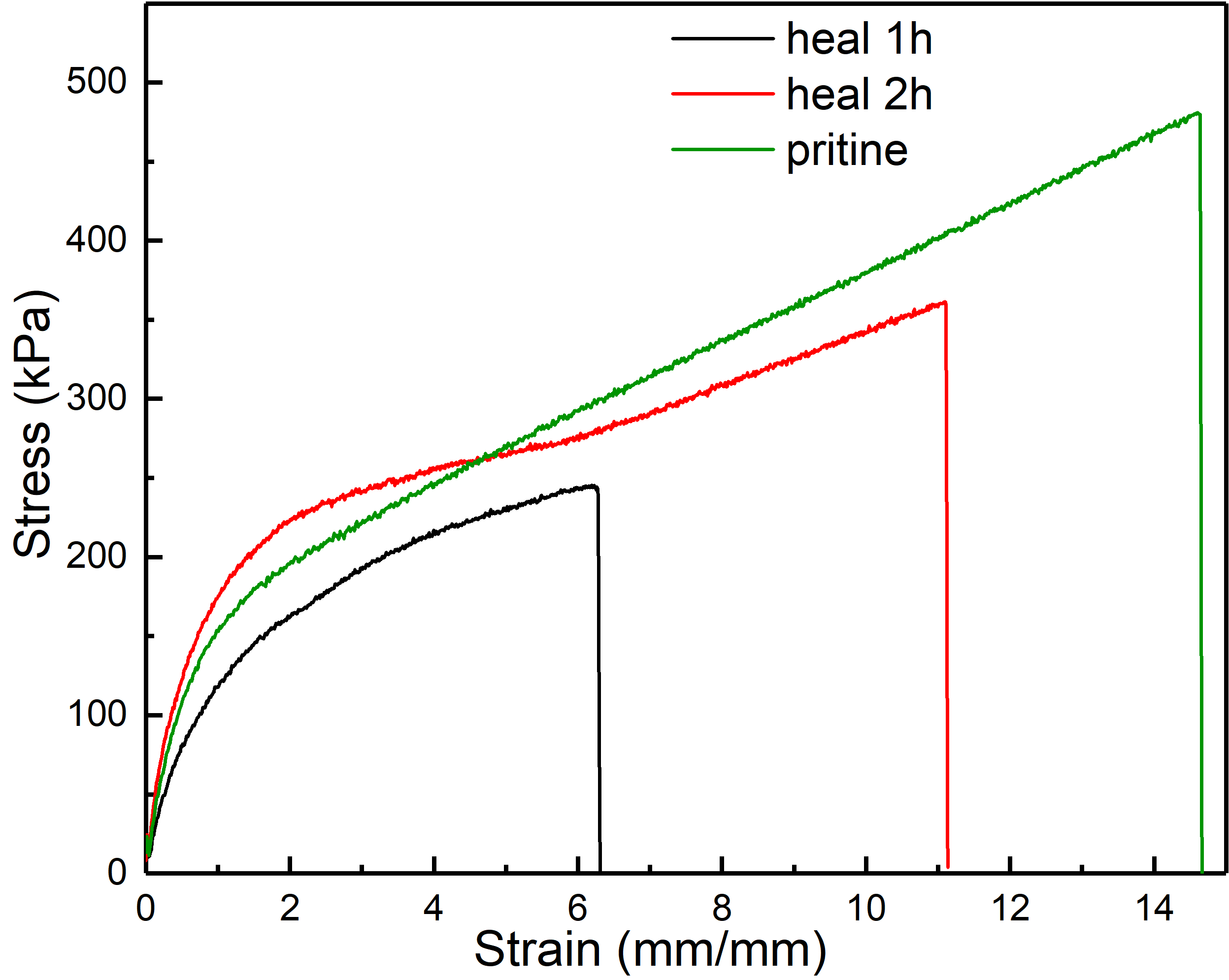


**Figure** **S5.** Stress-strain curves of TFPM-PDMS-25000 films healing for different times at 0 ^o^C.

**
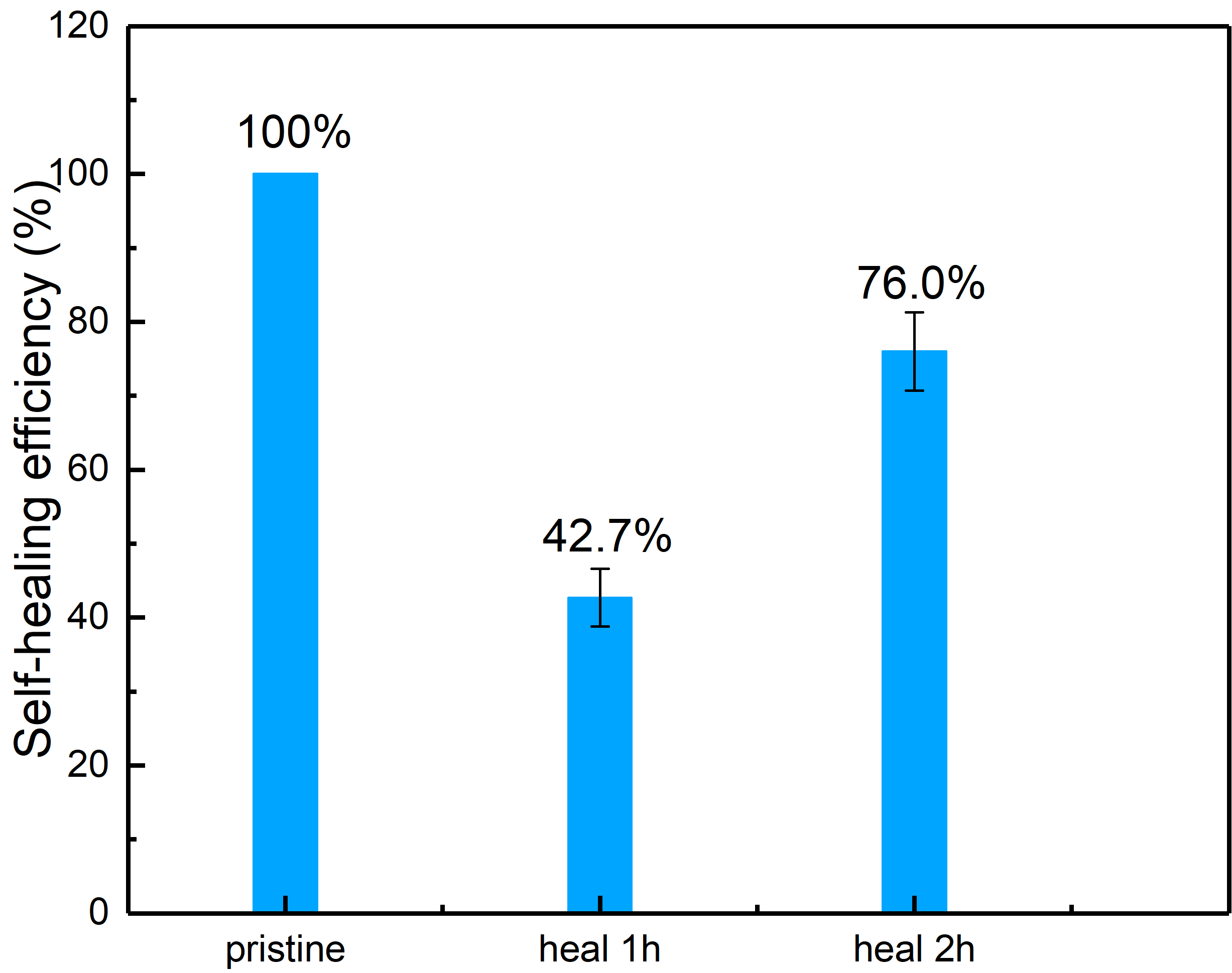
**

**Figure** **S6.** Healing efficiencies of TFPM-PEA-4000 with different healing durations at 0 °C.

**
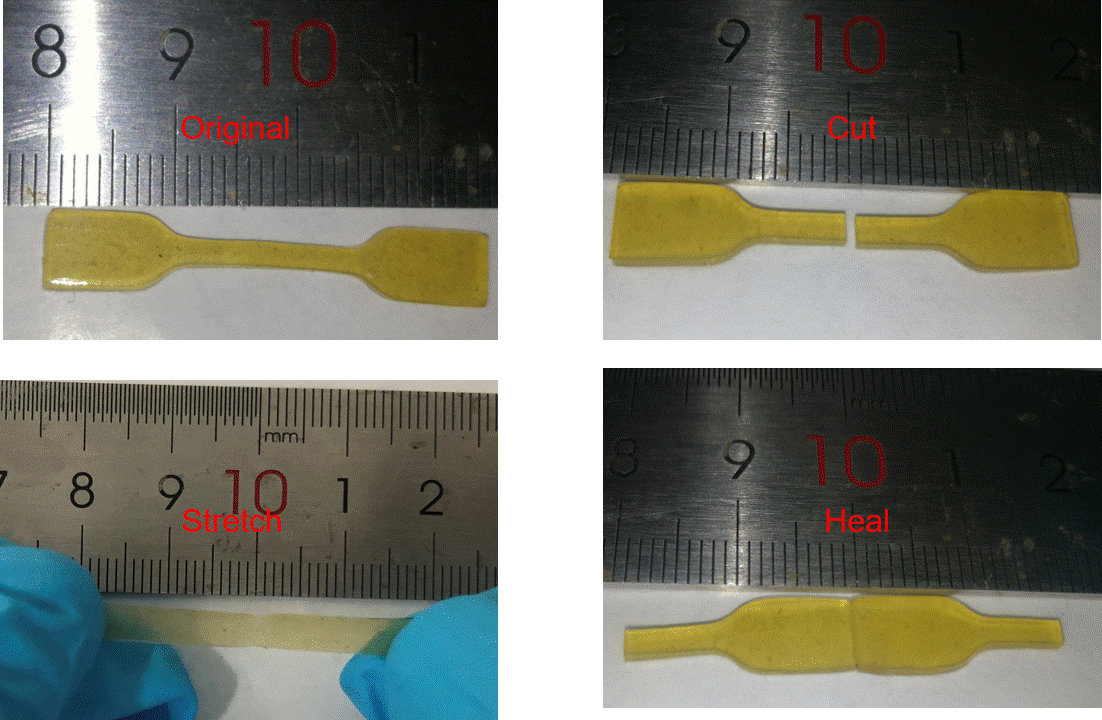
**

**Figure S7.** Photographs illustrating the macroscopic cutting-healing-stretching procedure of TFPM-PDMS-25000 films at 25 ^o^C.


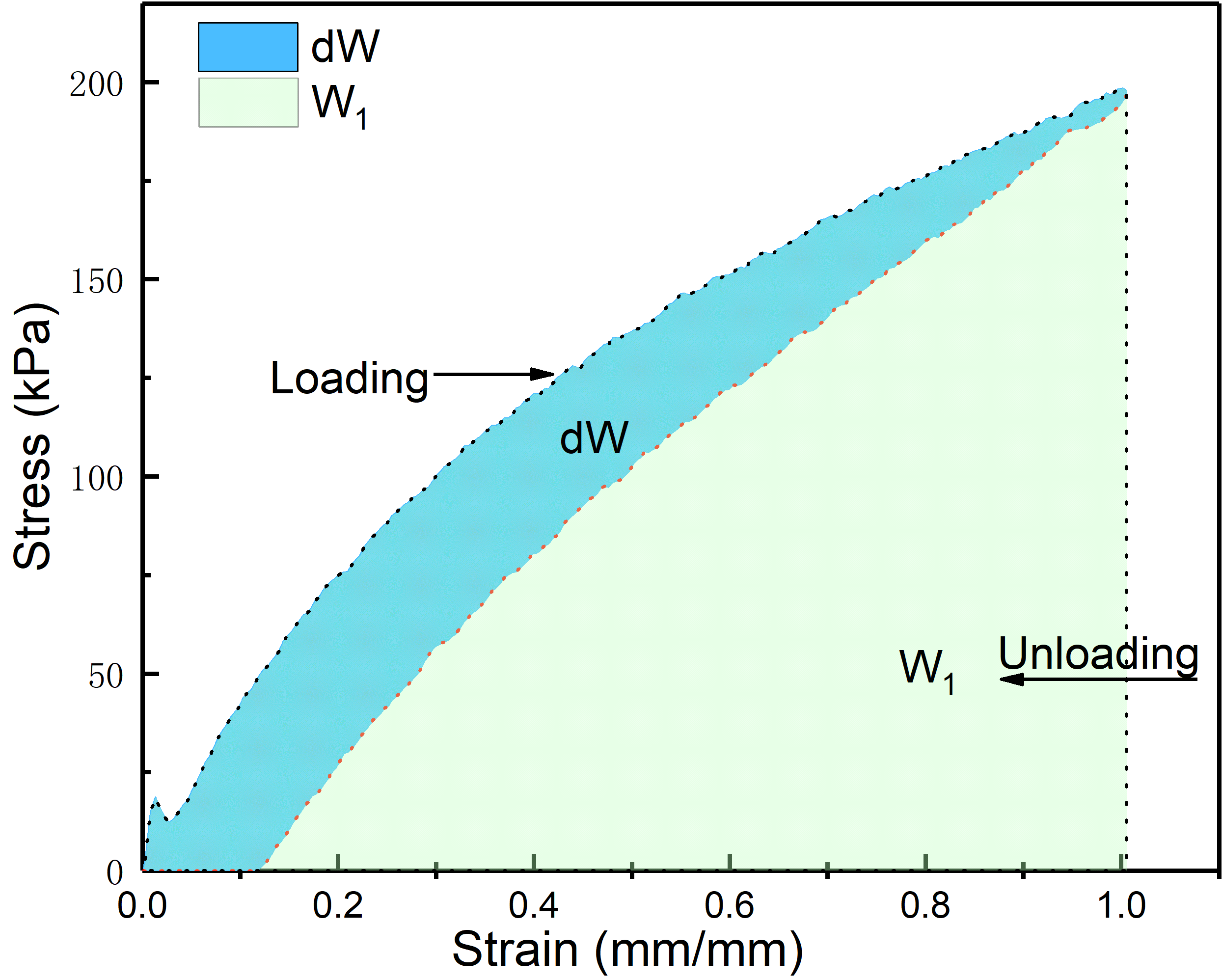


**Figure S8.** The energy absorbing properties of TFPM-PDMS-25000 polymer at 100% strain.

**Table S1**. Key Mechanical Properties of TFPM-PDMS-25000 Films

| Sample | Young’s modulus (kPa) | Elongation at break | Maximum tensile Strength (kPa) |
| --- | --- | --- | --- |
| TFPM-PDMS-25000 | 219.7 (± 6.2) | 1400 (± 200)% | 481.1 (± 8.4) |

**Table S2**. The energy absorbing efficiency ω of TFPM-PDMS-25000 for different strains at room temperature

| Strains  ω | 100% | 200% | 400% | 600% | 800% |
| --- | --- | --- | --- | --- | --- |
| ω | 23.4% | 26.4% | 42.1% | 48.1% | 49.7% |
